# The "Private Lunacy Committee" and the Commissioners in Lunacy

**Published:** 1856-07-01

**Authors:** 


### THE "PRIVATE LUNACY COMMITTEE" AND THE COMMISSIONERS IN LUNACY.

We have received a printed circular, published at the instigation of a "Private Committee," formed to protect the interests of those connected with private asylums for the treatment of the insane. It refers to an official communication made a few months back by the Commissioners in Lunacy to the proprietors of all the private asylums, requesting them to keep at their respective establishments for the inspection of the Commissioners in Lunacy at the time of their visitation, a list of the patients under care and treatment, *stating exactly the amount paid for their board and maintenance!* Before making the slightest comment upon the question at issue, we beg to say that we are in entire ignorance of the gentlemen connected with this "Private Committee," and, in fact, we had no knowledge of its existence, until we received, by post, the printed circular referred to. It would appear that the opinion of Her Majesty's Solicitor-General (Sir R. Bethel) has been taken on the point, and this able and distinguished lawyer is clearly of opinion that the Commissioners in Lunacy, in exacting such a return from the proprietors of private asylums, have exceeded the authority vested in them by the Act of Parliament under which they act. Sir R. Bethel, however, judiciously suggests that those connected with private asylums should respectfully protest against the course which the commissioners have adopted, and leave, for the present, the matter in their hands. We consider this excellent advice, and we hope it will have proper weight with those to whom it is addressed. We have no doubt that the Commissioners in Lunacy will, in deference to the opinions of one of the most accomplished and erudite lawyers of the day, either withdraw their circular altogether, or materially modify its requirements. It is certainly very desirable that the Commissioners should possess the power of ascertaining in certain cases whether a sufficient sum is allowed and appropriated for maintenance and medical care, and to this reasonable power no sensible man can raise an objection; but we must confess we cannot recognise the justice of the attempt to compel every proprietor of an asylum to expose his private pecuniary affairs to the inspection of the Commissioners in Lunacy, or any person deputed by them to officially inspect the private asylums for the insane existing in this country. It has the appearance of being an un-English and inquisitorial mode of procedure, and cannot be otherwise than objectionable to gentlemen of refined and sensitive feelings. We feel satisfied that the Commissioners, in carrying out the provisions of the law, are only influenced by the purest and most honourable intentions, and that they will, in deference to the opinion of the "Private Committee," re-consider their questions at an early meeting of the Board, and will (if they are satisfied that they have inadvertently exceeded the powers legally intrusted to them) at once meet the objections that have been raised, and withdraw the circular which has given such offence. We again state that the Editor of this Journal was not consulted in respect to the formation of this Committee, and does not even know the name of a single individual associated with it.

